# Sharing of Potential Nest Sites by *Etheostoma olmstedi* Males Suggests Mutual Tolerance in an Alloparental Species

**DOI:** 10.1371/journal.pone.0056041

**Published:** 2013-02-28

**Authors:** Kelly A. Stiver, Stephen H. Wolff, Suzanne H. Alonzo

**Affiliations:** 1 Psychology Department, Southern Connecticut State University, New Haven, Connecticut, United States of America; 2 Department of Ecology and Evolutionary Biology, Yale University, New Haven, Connecticut, United States of America; 3 Department of Economics, Rice University, Houston, Texas, United States of America; Columbia University, United States of America

## Abstract

When reproductive competitors tolerate or cooperate with one another, they may gain particular benefits, such as collectively guarding resources or attracting mates. Shared resources may be those essential to reproduction, such as a breeding site or nest. Using the tessellated darter, a species where males but not females compete over potential nest sites, we examined site use and sharing under controlled conditions of differing competitor density. Sharing was observed even when competitor density was low and individuals could have each occupied a potential nest site without same-sex sharing. Males were more likely to share a nest site with one other when the difference in size between them was larger rather than smaller. There was no evidence that female sharing was dependent on their relative size. Fish were generally more likely to use and share larger sites, in accordance with the greater relative surface area they offered. We discuss how one or both sharing males may potentially benefit, and how male sharing of potential nest sites could relate to female mating preferences. Tessellated darter males are known to provide alloparental care for eggs but this occurs without any social contact between the alloparent and the genetic father of the young. Thus, the suggestion that they may also share sites and maintain social contact with reproductive competitors highlights the importance of increased focus on the potential complexity of reproductive systems.

## Introduction

Territoriality and intrasexual competition are two well-examined areas of animal behavior, and research on competitive interactions has often focused on access to reproductive resources such as nest sites. However, transient or permanent collaborations resulting in mutual benefits can arise even in the presence of reproductive competition, such as when two competitors share a reproductive site. These alliances may involve mutual tolerance (choosing to accept the presence of the other rather than attempting to leave or drive them away), active collaboration against competitors, and partner preference (choosiness over which competitors to associate with) [1]. Interactions involving all three components are typically termed coalitions, and can be considered a form of cooperation [2]. In accordance with the increased potential benefits of sharing, tolerance or cooperation at nest sites is expected to be more common when breeding opportunities or essential reproductive resources are limited, and more frequently involve higher-value reproductive resources [3]–[12].

Tolerance and cooperation with competitors can allow individuals to mitigate the costs of continued competition, to increase their mating success, sometimes through joint mate attraction, or to jointly guard an essential resource, such as a nest site [3], [13]–[17]. Individuals can be selective over whom they ally with, and may make active partner choices based on the relative dominance rank and competitive ability of themselves and their potential partners. For example, forming a temporary partnership may be of the greatest benefit to two subordinates who are unlikely to independently secure a resource [15], [18]. Alternatively, subordinates may benefit by joining a dominant individual who can better attract potential mates [2], [14], [19], [20], or they may prefer a social group where they have a higher dominance ranking, even if they are not the most dominant [10]. In turn, dominants may preferentially pair with weaker individuals whom they can better dominate [21]–[24].

Compared to research in birds and mammals, alliances and cooperative coalitions in fishes have received relatively less attention ([25]; see [26]–[27] for an overview of the occurrence and forms of cooperation and mutual tolerance in fishes). While it has been argued that the life history of fishes may be less predisposing to the evolution of complex sociality, it has also been suggested that sociality in fishes may be generally overlooked, perhaps because it can initially appear less complex than that observed birds and mammals (where the focus of study is often on such complex systems as cooperative breeders, where subordinates care for the young of dominants and often show delayed reproduction and dispersal [25], [28], although this form of cooperation does occur, perhaps less rarely, in other taxa [25], [28]). Territory or nest sharing between reproductive individuals has been documented in some species where individuals also provide care for the young of their allies (alloparental care, e.g. *Julidochromis ornatus* [29]). Joint nest defense and tolerance of specific reproductive subordinates is observed in some territorial fishes, including those with complex alternative mating tactics (e.g. the ocellated wrasse, *Symphodus ocellatus* [30], [31]). Additionally, joint courtship has been documented in some species (e.g. the greenside darter, *Etheostoma blenniodes* [32]). We experimentally examined nest sharing in the tessellated darter (*Etheostoma olmstedi*), a congener to *E. blennoides*. These males are known to display alternative mating tactics (which, as noted above, in other species can involve mutual tolerance or cooperation), and also occasionally care for non-descendant young (alloparental care; which occurs without social contact between males [33]–[36]). As a result, examining potential sharing in *E. olmstedi* is a valuable addition to the general study of the evolution of sociality (see also [26]).

Tessellated darter males compete over access to both females and nests using one of three different reproductive strategies, two of which involve territoriality [33], [34]. Territorial males guard and spawn at nests, and the degree of parental care they provide varies with male size: larger territorial males spawn at and abandon nests, while smaller territorial males move into abandoned nests and care for existing eggs while spawning with females note that this alloparental care does not involve social contact between the genetic father and the adoptive father [33]; [34], [36]. Other smaller males sneak spawn as female mimics [33], [34]. Sneaking and nest abandonment both contribute to mixed brood paternity [35]. However, genetic data reveal that smaller territorial males also father a portion of the young that subsequently receive alloparental care [36], and we have on rare occasion observed group spawning and multiple territorial males apparently sharing a nest (about 5 and 2 observations of each respectively; KAS, SHA, pers obs). Thus, male nest sharing could also account for a portion of the documented mixed paternity, an interesting possibility in a species that is known to engage in non-social adoptive alloparental care. Using a controlled experiment, we examine whether males share nest sites, and if so, what factors influence sharing.

If male tessellated darters do share potential nest sites, it is unclear whether to expect that they be biased towards sharing with particular individuals. Male displacement at sites occurs in accordance with size (large males displace smaller males, often without any direct aggression), and active aggression is less common than such passive displacement ([33], [34] KAS, SHA pers obs). As female tessellated darters more commonly spawn with large males [37], sharing between larger/dominant and smaller/subordinate males may be most expected: large males can more easily displace smaller partners if necessary in the future, while smaller males may potentially gain from the attractiveness and defensive abilities of the largest males (more so than larger males who have inherently higher attractiveness and defensive abilities [15], [21], [33], [34], [36], [37]. Site sharing may also be a method by which subordinates increase their defensive ability and access to females, particularly if females prefer the presence of multiple males [15], [18], [38], in which case we might expect sharing to more commonly involve intermediate or smaller sized individuals (due to their decreased access to females based solely on their own size [37].

Our intention was not to determine whether tessellated darter males *prefer* to share nest sites (even if mutual tolerance or cooperation occurs, there is no basis to suggest it would be beneficial for most individuals to engage in it), but to document whether sharing does occur and under what circumstances. We examine whether male size rank relates to likelihood of sharing and with whom, whether the sharing rate appears to relate to competitor density (and therefore site availability, see [3]–[12]), and whether higher-value resources are more commonly shared (here a larger site; see [3]). In contrast to males, female tessellated darters occupy sites only transiently to evaluate males and spawn; they do not guard or compete over them [33], [34]. There is no evidence of reproductive competition between tessellated darter females, and selection likely favors female association: shared mating preferences lead females to spawn in the same locations, likely a result of the predation dilution benefits for offspring survival [34], [37], [39]. Females are often found together, both at and away from breeding sites. Thus, sharing among females (who are not expected to avoid one another) is examined primarily as a comparison to male sharing, and to track female position as an indication of the potential benefits of sharing for males.

## Methods

### Ethics statement

This study, including all protocols, methods, and design details (e.g. number of individuals used), was approved by and conforms to the ethical guidelines of Yale University (Institutional Animal Care and Use Committee Protocol #2008-10908), and was conducted with the permission and approval of the Connecticut Department of Environmental Protection, Inland Fisheries Division (scientific collecting permit number SC-08002).

### Study species

Tessellated darters breed under rocks or wood debris; distinctly colored territorial males guard nests and are the sole carers for young (coloration maintains crypsis and differs from the bright non-cryptic coloration seen in other darters [33], [40]). Females do not provide care for young and breed repeatedly throughout the season at various nests [41]. As outlined above, the mating strategies of male tessellated darters result in both cuckoldry- and adoption-based alloparental care [33], [34], [36]. The benefit of egg adoption arises as a result of female mating preferences for eggs with nests; thus, site abandonment and subsequent alloparental care does not necessitate social interaction between the two males ([33], [34], [36], [37], [39]).

In other species, there is evidence both for and against the role of relatedness in determining which reproductive competitors share territories [3], [14], [16], [19], [20], [42]–[53]. As the tessellated darter life history suggests little opportunity for continued association with relatives, and the mean relatedness between tessellated darter adults within a breeding population is very low [36], we consider relatedness to be an unlikely foundation for potential nest site sharing. For this reason, and because any effect is unlikely to be detected without a focused experiment using individuals of known relatedness, we did not examine the possible effects of relatedness between males here.

### Experimental set-up

Studies were conducted on-site between April and June of 2009 and 2010 at the Salmon River Fishway in Leesville CT (72°28′55″W, 41°30′42″N). Males with territorial coloration and gravid females were caught using hand-nets and brought to shore; fish not fitting these criteria were immediately returned to the stream. Fish were caught on natural or artificial nests, as reproductive darters during the breeding season are most often found at such nest sites (KAS, SHW, SHA, pers obs). Each fish was measured (standard length to the nearest 0.5 mm) and uniquely marked with either visible implant elastomer (Northwest Marine Technology, Inc) or non-toxic acrylic paint. Fish were handled for 1–2 minutes each, and otherwise kept in a constantly aerated 18.9 liter bucket filled with river water. Some individuals were secured in a bait bucket and held overnight in the stream (water cover and flow minimized temperature change and ensured oxygen and food availability) prior to their use in a trial. Fish were unharmed by marking and housing, and freed into to the river after their use in the study.

For each trial, a 62.4 liter Sterilite™ container measuring 31.1 H×41.3 W×59.7 L (cm) was filled 3/4ths with river water and partially submerged at the river's edge (to ensure consistent temperature). The tank bottom was filled with 1 cm of sand and gravel from the river bottom, and the lid placed on loosely in a manner that allowed unobtrusive removal. An air stone was placed in the center of the tank. Each tank was wrapped in shade-cloth or cotton netting to obscure the outside of the tank and mimic the light level in the river.

Nest sites were constructed from slate tiles, each with two 4.0 cm lengths of 2.5 cm diameter PVC piping attached to adjacent corners. Each tile sloped up, with one edge along the bottom of the tank. Tiles and natural nest sites are generally only used by darters as breeding sites during the breeding season (KAS, SW, SHA, pers obs), as their method of defense against predation relies on crypsis and freezing rather than shelter (note that whether tiles may be viewed as shelter has not yet been tested; KAS, SW, SHA, pers obs [40], [54], [55]). Presence of a male at a tile is predictive of later egg deposition at that tile, supporting our treatment of tiles as potential nest sites for tessellated darters [38]. Each tank had one large (14×28 cm, with the 28 cm edge placed along the gravel) and one small (10×10 cm) tile, placed along opposite short edges of the tank so that openings faced the center of the tank (for diagram of tank set-up, see supplementary figure S1 in file S1). The large tile had a greater surface area available for eggs (a feature known to be preferred by spawning tessellated darters [34], [37]).

To examine whether the availability of potential nest sites influenced site sharing, the three experimental conditions varied the density of fish, but not the adult sex ratio (1∶1 in all trials). Two (*N*  =  21 trials), 3 (*N*  =  20 trials), or 4 (*N* = 21 trials) each of both males and females were placed in the experimental tanks. Size differences between males ranged from 0.00–1.85 cm between males who were adjacent in the size hierarchy (mean difference  =  0.42 cm; absolute size ranged from 4.00 to 8.25), while size differences of females adjacent in the hierarchy ranged from 0.00–1.50 cm (mean difference  =  0.28 cm; absolute size ranged from 3.80 to 6.10). Due to the number of fish needed and the time and condition restrictions on catching, some fish were reused once, but any two fish were never paired together in more than one trial, and 24 hours passed between any two trials in which an individual fish participated. In total, 110 males and 135 females were used.

All fish for a trial were simultaneously released into the center of the tank by allowing them to swim out of a partially submerged 1-litre plastic container. Fish were observed at one-hour intervals for 4 hours (4 observations total; three trials had only three observations on one or all fish. An incoming storm necessitated early termination of two trials, and the location of one individual was not determined due to recording error for one check of a third trial). This frequency of checks was chosen to minimize the disruption of fish positions. For each observation, the lid of a tank was lifted and set aside, and the identity of any fish not under a tile (termed “out”) recorded. Then, each tile was slowly lifted (the bottom edge remained in contact with the bottom of the tank) and the identity of each fish under it noted before the tile was placed back into position. Darters tend to remain in place during such checks [37], [39]. The order in which tiles were lifted was alternated between observations. After all fish positions were recorded, the lid was replaced. At the conclusion of a trial, the underside of each tile was examined to note egg deposition, and photographed using an Olympus Tough8000 or Sw770 camera. Eggs were not collected nor were paternity analyses conducted, as there is low DNA yield from eggs that are only a few hours old (KAS, SHW, pers obs), and we could not house the eggs for further development.

### Statistical analyses

We planned our analyses to remove the potential effect of pseudoreplication in the dataset (as paired comparisons resulted in each fish contributing to multiple data-points and multiple observations per trial, and there was an occasional re-use of individuals, although never of pairs). We controlled for non-independence by averaging fish position across checks to determine under which tile each individual spent the majority of the observations, and using randomization tests wherever applicable (Rundom Pro 3.14 with 10,000 randomizations [56], for further discussion see [57]). Additional tests were conducted using SPSS 19.0, and all tests were two-tailed. Due to recording error, we have only relative but not absolute size differences for two females; these females are excluded from analyses requiring absolute size.

We report the overall rates of sharing for males and females, and test for an effect of site availability. For clarity, the majority of female patterns, particularly those that mirror findings for males, are reported in supplementary materials except where noted and where they inform or contrast with male patterns (see supplementary text, supplementary tables S1 and S2 in file S1). Whether individuals were selective of who they shared with (a potential indicator of partner preference) was examined by determining how sharing related to individual size (both absolute, and relative based on the body size ratio of the two individuals [58]), individual size rank (based on the sex-specific size-rankings), and the influence of density on sharing between fish of different size ranks. We compared male and female position in the tanks to determine whether the position of one sex related to the position of the other. We also examined whether the more valuable site (the large tile, which offers greater potential spawning area) was preferred and shared more often, as well as the effect of individual size and size rank on its use. Finally, we examined the rate of switching between tank areas (out from under tiles, under large tile, or under small tile) to determine whether males and females differed in their switching rate, and if this rate differed based on individual size, size rank, or experimental condition.

## Results

### The influence of potential nest site availability on sharing

**Figure 1:**
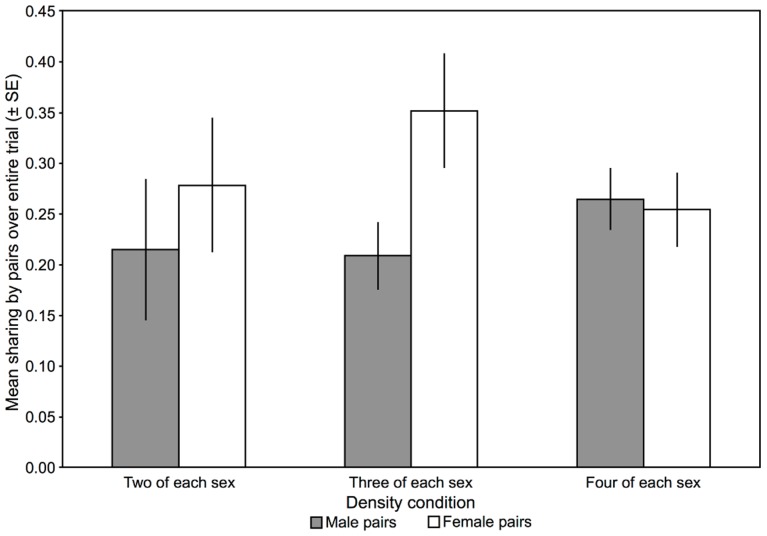
Rate of sharing between same-sex pairs. Density condition did not influence the rate of sharing between same-sex pairs, although there was a trend suggesting that females may be more likely to share with one another than males are (based on the proportion of checks for which a particular pair was found to be sharing a tile; repeated-measures ANOVA, sex: *F_1,59_*  =  3.13, *p*  =  0.08; density condition: *F_2,59_*  =  0.17, *p*  =  0.84; sex×density condition: *F_2,59_*  =  1.77, *p*  =  0.18). As the lowest density condition had the same number of territories as it had same-sex individuals, each individual could have had his or her own potential nest site without sharing with a fish of the same sex if they would show maximum outspacing. The sharing observed suggests that site availability did not underlie sharing.

While there was a trend suggesting that females shared with one another more often than males did, there was no influence of density condition on sharing rates (see figure 1). This suggests that the availability of potential nest sites does not underlie sharing, as in the lowest density condition there are sufficient tiles for each same-sex individual to occupy a tile without sharing.

### Size and sharing with same-sex fish: evidence for partner preference

#### i) Sharing based on absolute and relative size

**Figure 2:**
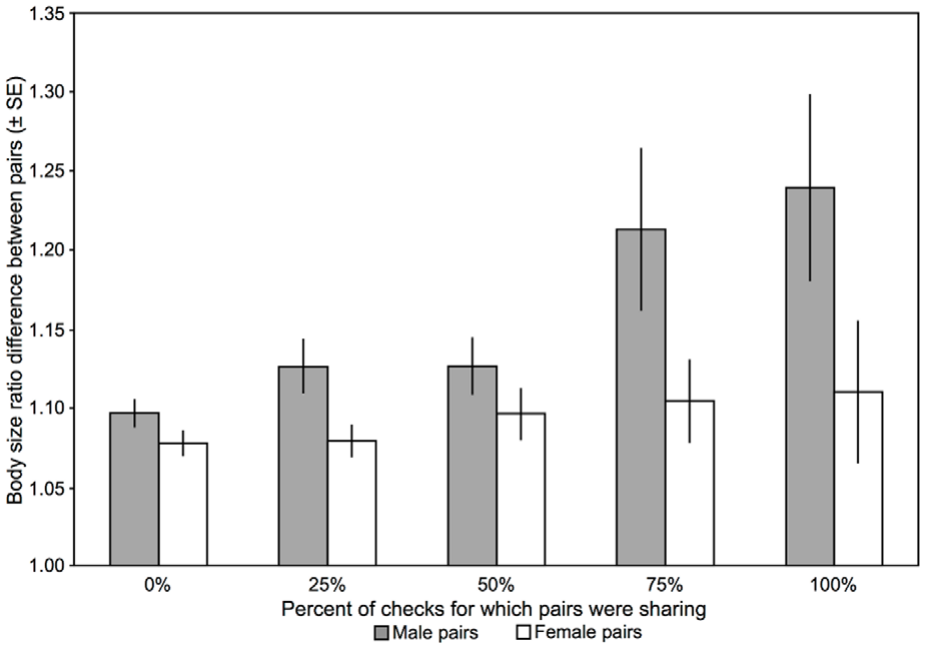
Rate of sharing and difference in relative size between same-sex pairs. Mean body size ratio difference ± SE for same-sex pairs of males (in grey) and females (in white) grouped by whether they were sharing a tile for 0, 25, 50, 75 or 100% of the four checks of a replicate (excluding trials for which only three checks could be completed). The differences between groups mirror correlational analyses (see text): sharing among males was more commonly observed when males were more different in size, whereas there was no relationship between difference in size and proportion of the checks for which female pairs shared a tile (One-way ANOVA, randomization version, males: *F*_4,198_  =  5.19, *p*  =  0.002; post-hoc (two-sample randomization tests with Holm's correction): pairs that shared on no checks were closer in size than those who shared on three (p  =  0.03) or four (0.005) checks ; females: *F*_4,194_  =  0.71, *p*  =  0.57).

Absolute size of males did not relate to their mean rate of sharing with other males (Ordinary least-squares regression with randomization test for slope  =  0, *r*  =  0.07, *N*  =  186, *p*  =  0.34; because this and additional tests of absolute size showed no significant results, tests of absolute size are not further reported here). However, males who were more different in size had a higher rate of sharing (body size ratio of the pair: *r*  =  0.280, *N*  =  207, *p*  =  0.0002; figure 2), and there was a trend suggesting that male pairs not adjacent in the size hierarchy shared a tile more often than males that were adjacent in the size hierarchy did (Two-sample randomization test, *test stat* = −0.077, *N*  =  124, 83, *p*  =  0.053). Similar to males, absolute size of females did not relate to their mean rate of sharing with other females (*r*  =  0.03, *N*  =  184, *p*  =  0.71; again, tests of absolute size are from this point omitted). However, neither differences in size nor adjacency in the size hierarchy related to the rate at which pairs of females shared (body size ratio of the pair: *r*  =  0.109, *N*  =  203, *p*  =  0.12; figure 2; adjacency: *test stat*  =  0.011, *N*  =  123, 81, *p*  =  0.79). Thus, males are more likely to share with those males most different from them in size, regardless of absolute size. In females, the rate of sharing is independent of their relative and absolute size.

#### ii) Sharing based on size rank

There is no evidence that males' size rank related to how often they shared a tile (One-way randomized ANOVA, *F*_3,182_  =  1.59, *p*  =  0.19). Density did not relate to the rate of sharing between males of particular size ranks, or the rate of sharing between the largest and smallest males (table 1; for female results, see supplementary table S1 in file S1).

**Table 1:** Comparisons of sharing by males of different size ranks.

Comparison	Test statistic	*p*-value
Size ranks 1 and 2	*F* = 0.04	0.96
Size ranks 1 and 3	*t* = 0.74	0.46
Size ranks 2 and 3	*t* = −0.52	0.61
Largest and smallest	*F* = 1.18	0.31

Same-sex sharing between males of specific size ranks was not influenced by the density condition. Tests are one-way ANOVAs or unpaired *t*-tests (test statistic indicated). Sample sizes for each density condition were: four-fish, *N*  =  21; six-fish, *N*  =  20; eight-fish, *N*  =  21. Included size rank pairs are only those present in more than one condition.

### Intersexual sharing

The mean number of males and females in the three possible tank positions for each trial was correlated (under large tile, under small tile, out from tiles), suggesting that choice of position was not independent between the sexes (Ordinary least-squares regression with randomization test for slope  =  0, *r*  =  0.47, *N*  =  204, *p*  =  0.0001). Correlated positions of males and females could result from proportionate usage of space by each sex, mutual location preference, or preference for being with fish of the other sex. More females were under tiles that males were sharing (*N*  =  131 tile observation) than were under tiles males were not sharing (*N*  =  359 tile observation; included is each female's position for each observation of each trial, two-sample randomization test, *test stat*  =  0.21, *p*  =  0.001).

### Sharing and value of the sites

#### i) Use of tiles, and of large versus small tiles

The mean proportion of checks for which individual males were out from under the tiles was 0.21. 168 males spent the majority of checks either out from tiles, or under either tile. Of these, 140 were most often under a tile and 28 out from under tiles; thus, males were more often using tiles than they were out from them (binomial test, probability under tile  =  0.199 (area of tank covered by tiles), 140/168, *p* < 0.0001). To determine whether males showed biased usage of either the large or small tile, a “preferred” tile was determined for 161 males who were under either the large *or* small tile for the majority (3 or 4) of the checks. Of these, 114 males were more often under the large tile. Within trials, males were more often under the large tile than under the small tile (one-sample *t*-test, *t*  =  8.43, *N*  =  62, *p* < 0.0001), although males used the small tile more than would be expected based on random choice (based relative tile size; binomial test, probability under large  =  0.797; 114/161, *p*  =  0.01). Regardless, the large tile was more commonly shared than the small tile, and at a significantly higher rate than expected based on random chance (counts of how often each was shared out of the total number of check during which tile sharing occurred: binomial test, probability under large  =  0.797; 114/128, *p*  =  0.008). Thus, while males were more likely than chance to *use* the small tile (but still more likely to use the large overall), they were more likely than chance to *share* the large tile. Females were more often under the large tile, used each tile with the probability that would be expected based on the relative size of the tiles, and also shared the large tile significantly more often (see supplementary text in file S1).

#### ii) Effect of relative size on use of tank areas and large versus small tile

We tested whether males of different size ranks showed a bias in how much they used the tiles, or in using the large versus small tiles. Males were on average under tiles more often than would be expected by chance (based relative surface area of tiles versus the entire tank; see table 2a). However, when only those checks where an individual used a tile were considered, we found that only the largest and smallest males (size ranks 1 and 4) were using the tiles as would be expected by random chance: males of size rank 2 and 3 were less likely to be under the large tile than would be expected based on the area which it covered relative to the small tile (see table 2b; all females used the large tile at the rate expected by chance, see supplementary table S2 in file S1). This pattern of tile usage is consistent with what we would expect if large males control tile residency and only tolerate males that are relatively smaller than they are and is the likely cause of the increased usage of the small tile noted above.

**Table 2:** Comparisons of preferred position in the tank.

Individual Size Rank	A) Number who used the tiles (versus remaining out)		B) Number who used the large tile (versus small tile)	
	Under tile∶Out	*p*	Large∶Small	*p*
Male 1	48∶8	**<0.0001**	41∶13	0.59
Male 2	47∶9	**<0.0001**	34∶17	**0.04**
Male 3	30∶7	**<0.0001**	21∶14	**0.012**
Male 4	15∶4	**<0.0001**	18∶3	0.71

The random chance of using a location was considered as the percentage of the total involved area (therefore, tiles represents 19.9% of the total tank area, and the large tile represents 79.7% of the “under tile” area in a tank). Binomial tests considering both methods of determining “random” placement were run. All p-values are two-tailed.

a) Males were more likely than expected by chance (based on the area of the tank covered by tiles) to spend a majority of checks under the tiles rather than out.

b) While the largest and smallest males used the large and small tiles as expected based on random usage (determined by the area covered by each tile), males of size rank 2 and 3 used the large tile less than would be expected by chance.

Note: the numbers between comparisons vary, since males could only be included in these analyses if a preference could be established based on their being in a particular location for the majority of either all checks (a) or those checks for which they were under any tile (b).

#### iii) Egg placement on tiles

When eggs were found, they were most often under the large (*N*  =  9) rather than the small (*N*  =  3) tile, though in one trial, eggs were found under both tiles. Although the number of observations is insufficient for statistical testing, this observation mirrors the above findings suggesting that tessellated darters use nests in accordance with the surface area they offer.

### Movement within the tank (switching location)

Females switched their location in the tank more often than males did (individuals and trials with only three observations excluded, mean ± SE; males  =  1.04±0.10; females  =  1.30±0.08; paired t-test, *t*  =  2.20, *N*  =  60, *p*  =  0.03). However, the switching rate did not relate to individual size or size rank, or to the average size differences between individuals in a trial, or to trial condition (see supplementary text in file S1).

## Discussion

Our findings confirm that tessellated darters do share nest sites with same-sex conspecifics, with females perhaps sharing more often than males. The rate of sharing by both sexes did not appear to be influenced by density, contrary to the expected role of ecological constraints in nest site sharing (see [3]–[5], [7]). This is notable, as alloparental care in this species appears to be more common in areas with high nest availability, also contrary to general expectations [36]. For both males and females, absolute size did not affect sharing rates. Males appeared to be selective over whom they shared with, and sharing was most common between males that were more different from one another in size. It also appeared that sharing was less common between males who were adjacent in size rank. However, this did not translate to consistent sharing by males of particular size ranks. In short, male sharing appears to depend on relative details of a particular male pair, not absolute features of the individuals. In contrast, females did not exhibit any evidence of preferring or avoiding other specific females, as expected based on the life history of the species and known mating preferences of female darters (which suggest low female-female competition and frequent female grouping; [33], [34], [36], [37], [39]). The increased movement of females around the tank relative to males is consistent with males being more territorial than females.

In general, although darters appeared to prefer to be under rather than out from tiles, males overall used the small tile more often than random chance would predict, likely the result of increased usage of the small tile by males of size ranks 2 and 3. However, the large tile was *shared* more often, and at rates greater than expected based on random usage of tiles due to relative tile size. This differential use of the large tile is in line with male sharing based on their relative size to one another, and suggests that the largest males may selectively exclude others that are too close to them in size. Females showed no bias in tile usage, but also shared the large tile a rate higher than expected by random usage based on the relative size of each tile.

Our findings suggest that the apparent site sharing by tessellated darter males may represent a more socially complex interaction than simple mutual tolerance, as there is suggestion of preference for sharing between males who were more different in size. Partner preference has been put forth as a trait of cooperative coalitions, a form of social interaction more complex than mutual tolerance [1]. As the size-specific sharing we observed was the product of an unseen associative process, we intend only to suggest that males may actively seek or prefer particular partners; confirmation of a true partner preference (and therefore of the degree of complexity of this social interaction) is still needed. Regardless of the origin of the pattern, our observation of increased sharing by males who were more different in size suggests an alternate basis for genetic findings that smaller territorial males gained reproduction in the initial spawning phase at tiles that were being guarded by larger males [26]. Although we know that sneaking contributes to this paternity loss [33], [34], our results reported here suggest that transient site sharing between larger and smaller territorial males could contribute to the mixed paternity observed in other studies [36].

It is not yet clear whether and how males may benefit by sharing potential nest sites with other males. One possibility is that tolerance of sharing could result in decreased costs relative to those that males would experience if they continued active aggression to drive away a competitor. We would expect to see the observed pattern of more sharing between males based on their relative size if tolerance of a competitor mitigates the potential costs of sustained defense for larger males, and if larger males can reduce the possibility of later competition with males closer to them in size by accepting the presence of a weaker competitor. For a smaller male, the presence of the larger male could allow him to avoid additional competitors. However, if darters assess the number of additional competitors present in their immediate area (a possibility, given patterns of movement such as those documented in this study), this latter point is not consistent with our observation that sharing occurs even when only two males (and two territories) are present. Finally, sharing could reflect active cooperation, allowing males to increase their defensive ability against further competitors. It is unclear whether there is joint defense or courtship by the male pair, although this has been documented in the congener *Etheostoma blenniodes* [32], and further work is planned to examine this possibility.

Female mating preferences could also contribute to male site sharing. If females prefer tiles with a greater number of guarding males, sharing would confer a direct benefit to both males. Also, small males may benefit more by exploiting the attractiveness of a larger male rather than attempting to attract females on their own (see [39]). As described above, large males in this scenario could decrease their lost mating opportunities by tolerating other males and attending to interested females rather than actively defending. As the number of males and females under a tile are correlated, female choice may be responsible for this male site sharing, a possibility in line with the suggestion that the evolution of mutual tolerance/cooperation at nest sites may be best understood by considering limitations on breeding opportunities [7]. More work specifically examining individual movement and interaction of the males is needed to uncover the potential benefits of mutual tolerance (and potential cooperation) between tessellated darter males.

It is tempting to suggest that the previously documented alloparental care could actually be parental care by a smaller, sharing male following nest abandonment by the larger male. However, behavioral observations clearly reveal adoption of eggs, and genetic analyses confirm that alloparents often have no paternity of the young they are guarding [36]. The observation of site sharing by tessellated darter males suggests that there is potentially a second cooperative route to increased reproductive success available to smaller territorial males: these males may use both non-social potential cooperation (the nest abandonment/alloparenting exchange) and social interactions (transient site sharing) to increase their access to females and hence their reproductive success relative to what they could gain by independently guarding an empty nest [33], [34], [36], [37], [39].

While site sharing is often found in species with alloparental care [3]–[11], what is unique here is that the two behaviors are apparently independent. In fact, allocare in this species is generally the result of adoption following nest abandonment and generally does not involve social contact between the true and adoptive father of the young [33], [34] unlike other species where allocare results from piracy by dominants (e.g. the fathead minnow, *Pimephales promelas* [59]–[62]). The suggestion of another potential form of cooperation in the tessellated darter supports the idea that there should be greater attention to both less-examined forms of potential cooperation, and to species that have traditionally received less focus (e.g. fish compared to birds and mammals; see [25], [63]–[67]. Further work on female choice, and the specifics of the formation and actions of sharing males, will continue to illuminate sociality in this and similar species.

## Supporting Information

File S1 Contains supporting figures, tables, and text. **Figure S1: Schematic of the tank set-up used in this study.** Pictured is an A) top-down, and B) side view of the tank set-up used. All objects are scaled to their correct relative sizes. Tanks were 31.1 H×41.3 W×59.7 L (cm). AS  =  air stone, ST  =  small tile (10×10 cm), LT  =  large tile (14×28 cm). The curved line in picture B indicates water height. **Table S1: Comparisons of sharing by females of different size ranks.** Same-sex sharing between females of specific size ranks was not influenced by the density condition. Tests are one-way ANOVAs or unpaired *t*-tests (test statistic indicated). Sample sizes for each density condition were: four-fish, *N*  =  21; six-fish, *N*  =  20; eight-fish, *N*  =  21 (*N*  =  20 for comparison of largest and smallest females due to missing size data; see methods). **Table S2: Comparisons of preferred position in the tank.** The random chance of using a location was considered as the percentage of the total involved area (therefore, tiles represents 19.9% of the total tank area, and the large tile represents 79.7% of the “under tile” area in a tank). Binomial tests considering both methods of determining “random” placement were run. All p-values are two-tailed. a) Females were more likely than expected by chance (based on the area of the tank covered by tiles) to spend a majority of checks under the tiles rather than out. b) Females use of the large tile rather than the small tile did not differ from the usage expected by chance (based on the relative surface area of the tiles).
(DOCX)
