# Supplementary material for: Sharing of Potential Nest Sites by Etheostoma olmstedi Males Suggests Mutual Tolerance in an Alloparental Species
Source: PLoS One. 2013 Feb 28;8(2):e56041. doi: 10.1371/journal.pone.0056041 (PMC3585326; doi:10.1371/journal.pone.0056041)

**Supplementary Material**

*Results for sharing between female tessellated darters*

*ii) Sharing based on size rank*

As in males, there is no evidence that females’ size rank related to their rate of sharing (One-way randomized ANOVA, *F*_3,181_ = 0.50, *p* = 0.69). Density did not relate to the rate of sharing between females of particular size ranks, or between the largest and smallest females (see table S1).

*Sharing and value of the reproductive resource*

*i) Use of tiles, and of large versus small tiles*

The mean proportion of checks for which individual females were out from under the tiles was 0.32 respectively. 145 females spent the majority of checks either out from tiles, or under either tile. Of these females, 102 were most often under a tile, and 43 out from under tiles, demonstrating that females prefer to use tiles than be out from under them (binomial test, probability under tile = 0.199 (area of tank covered by tiles), 102/145, *p* < 0.0001). By excluding the checks for which individuals were out, we could determine a preferred tile for 136 females who were under either the large or small tile for most of the checks. Of these, 114 females were more often under large tile. Within trials, females were more often under the large tile than under the small tile (one-sample *t*-test, *t* = 9.84, *N* = 62, *p* < 0.0001) and the large tile was more commonly shared than was the small tile. Females used the large and small tiles at the rate expected by random choice (that is, relative tile size; binomial test, probability under large = 0.797; 114/136, *p* = 0.274). The large tile was more commonly shared than the small tile, and at a significantly higher rate than expected based on random chance (counts of how often each was shared out of the total number of check during which tile sharing occurred: binomial test, probability under large = 0.797; 124/139, *p* = 0.004). Thus, while females used the tiles at the rates expected by chance, they more commonly shared the large tile.

*ii) Effect of relative size on use of tank areas and large versus small tile*

To examine the effect of relative size, we tested whether females in different positions in the sex-specific size hierarchy showed an overall difference in whether they used the tiles, and in their use of the large and the small tiles. While females were under tiles more often than would be expected based on the area of the covered by tiles (see table S2a), females of all size ranks used the large and small tiles in accordance with random chance based on the relative size of these two tiles (see table S2b).

*Movement between tank areas*

Individual size and size rank did not relate to how often fish switched between tank areas for either males or females (Ordinary least-squares regression with randomization test for slope = 0; male size: *r* = 0.080, *N* = 181, *p* = 0.28; male size rank: *r* = 0.118, *N* = 175, *p* = 0.12; female size: *r* = 0.024, *N* = 180, *p* = 0.75; female size rank: *r* = 0.070, *N* = 161, *p* = 0.38). The mean number of switches per trial did not relate to the mean size difference of same-sex individuals in a trial (males: *r* = 0.011, *N* = 60, *p* = 0.94; females: *r* = 0.073, *N* = 59, *p* = 0.58), nor to the experimental condition (One-way randomized ANOVA, males: *F_2_*_,57_ = 1.56, *p* = 0.22; females *F_2_*_,57_ = 1.39, *p* = 0.25).

**Table S1: Comparisons of sharing by females of different size ranks.**

Same-sex sharing between females of specific size ranks was not influenced by the density condition. Tests are one-way ANOVAs or unpaired *t*-tests (test statistic indicated). Sample sizes for each density condition were: four-fish, *N* = 21; six-fish, *N* = 20; eight-fish, *N* = 21 (*N* = 20 for comparison of largest and smallest females due to missing size data; see methods).

| Comparison | Test statistic | *p*-value |
| --- | --- | --- |
| Size ranks 1 and 2 | *F* = 0.71 | 0.49 |
| Size ranks 1 and 3 | *t* = 1.71 | 0.10 |
| Size ranks 2 and 3 | *t* = 1.06 | 0.30 |
| Largest and smallest | *F* = 0.09 | 0.91 |

**Table S2:** **Comparisons of preferred position in the tank.**

The random chance of using a location was considered as the percentage of the total involved area (therefore, tiles represents 19.9% of the total tank area, and the large tile represents 79.7% of the “under tile” area in a tank). Binomial tests considering both methods of determining “random” placement were run. All p-values are two-tailed.

1. Females were more likely than expected by chance (based on the area of the tank covered by tiles) to spend a majority of checks under the tiles rather than out.
2. Females use of the large tile rather than the small tile did not differ from the usage expected by chance (based on the relative surface area of the tiles).

| Individual Size Rank | A) Number using the tiles (versus remaining out) | | B) Number using the large tile (versus the small tile) | |
| --- | --- | --- | --- | --- |
|  | Under tile:Out | *p* | Large:Small | *p* |
| Female 1 | 42:11 | **<0.0001** | 39:8 | 0.73 |
| Female 2 | 34:13 | **<0.0001** | 42:5 | 0.13 |
| Female 3 | 17:10 | **<0.0001** | 25:4 | 0.54 |
| Female 4 | 9:9 | **0.005** | 8:5 | 0.21 |

Note: the numbers between comparisons vary, since females could be included in these analyses if a preference could be established based on their being in a particular location for the majority of either all checks (a) or those checks for which they were under any tile (b).

**Figure S1**


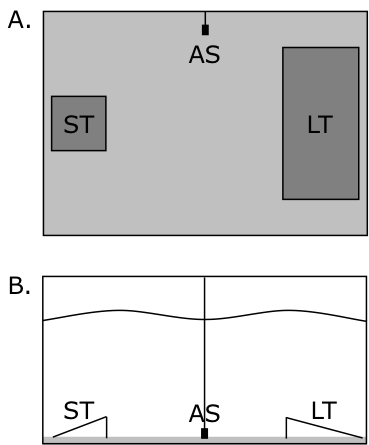

Supplement: File S1 — Contains supporting figures, tables, and text. Figure S1: Schematic of the tank set-up used in this study. Pictured is an A) top-down, and B) side view of the tank set-up used. All objects are scaled to their correct relative sizes. Tanks were 31.1 H×41.3 W×59.7 L (cm). AS = air stone, ST = small tile (10×10 cm), LT = large tile (14×28 cm). The curved line in picture B indicates water height. Table S1: Comparisons of sharing by females of different size ranks. Same-sex sharing between females of specific size ranks was not influenced by the density condition. Tests are one-way ANOVAs or unpaired t-tests (test statistic indicated). Sample sizes for each density condition were: four-fish, N = 21; six-fish, N = 20; eight-fish, N = 21 (N = 20 for comparison of largest and smallest females due to missing size data; see methods). Table S2: Comparisons of preferred position in the tank. The random chance of using a location was considered as the percentage of the total involved area (therefore, tiles represents 19.9% of the total tank area, and the large tile represents 79.7% of the “under tile” area in a tank). Binomial tests considering both methods of determining “random” placement were run. All p-values are two-tailed. a) Females were more likely than expected by chance (based on the area of the tank covered by tiles) to spend a majority of checks under the tiles rather than out. b) Females use of the large tile rather than the small tile did not differ from the usage expected by chance (based on the relative surface area of the tiles). (DOCX) [file pone.0056041.s001.docx]
